# Primary clear cell sarcoma of humerus: case report

**DOI:** 10.1186/1477-7819-9-163

**Published:** 2011-12-09

**Authors:** Xudong Liu, Huizhen Zhang, Yang Dong

**Affiliations:** 1 Department of Orthopaedic Surgery, Shanghai Sixth People's Hospital Affiliated to Shanghai Jiaotong University, Shanghai 200233, China; 2 Department of Pathology, Shanghai Sixth People's Hospital Affiliated to Shanghai Jiaotong University, Shanghai 200233, China

**Keywords:** Clear cell sarcoma, humerus

## Abstract

We report a case of primary clear cell sarcoma occurring in the humerus. A 20 year old girl was referred to our hospital two years ago with painless swelling of the soft tissue surrounding the proximal right humerus. The conventional radiographic image showed an ill-defined, aggressive, osteolytic lesion associated with a partial cortical defect of the proximal right humerus. Magnetic resonance imaging found an irregularly shaped extraosseous mass extending from the proximal shaft of the right humerus, penetrating through the destroyed bone and invading the biceps brachii. After preoperative chemotherapy, a total tumor resection, internal fixation and bone cement implantation were performed. Histological studies of the resected tumor found that fibrous septa divided into well-defined nests and polygonal and fusiform cells with clear cytoplasm. Immunohistochemical studies demonstrated strong positive cytoplasmic staining with S-100 and scattered positivity with HMB-45. These findings led to a final, definitive diagnosis of clear cell sarcoma of the humerus. After routine postoperative chemotherapy, a 2-year follow-up showed no metastasis. Our findings in this report point out that primary clear cell sarcoma can originate from within the humerus and that limb salvage surgery can obtain a good result.

## Background

Clear cell sarcoma is a malignant soft tissue neoplasm first described by Enzingerin 1965 [1]. It is rare, accounting for less than 1% of soft tissue sarcomas. It is even rarer occurring in bone. To our knowledge, only six cases of primary clear cell sarcoma of the bone have been reported in English literature [2-4], but none of them presented that the neoplasm arose from the humerus. This paper reports on a case of primary clear cell sarcoma that arose from the right humerus of a 20-year-old girl.

## Case presentation

A 20-year-old girl was admitted to our hospital with painless swelling in the proximal right humerus of 6 weeks' duration.

**Figure 1:**
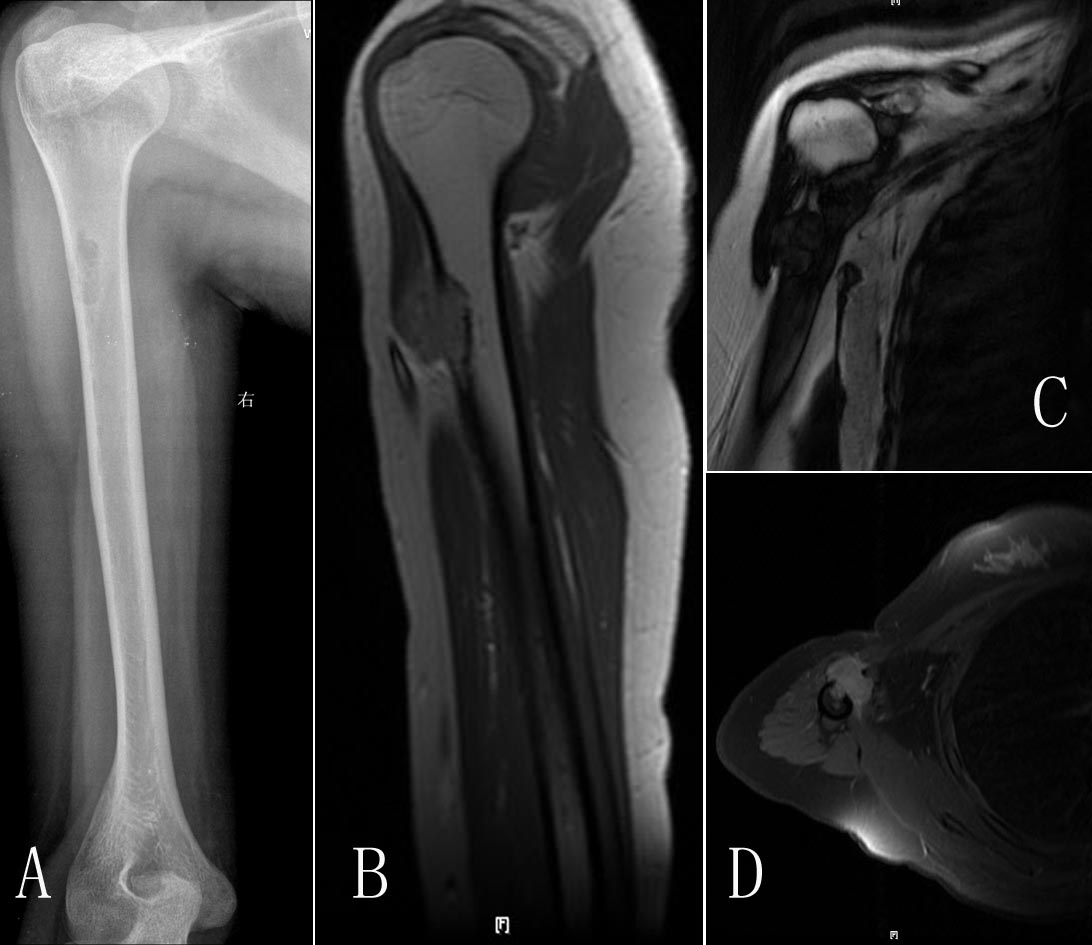
**A, Radiographs show an ill-defined, destructive, osteolytic lesion of the right proximal humerus**. B, C, D T1, T2 and axial view MR images show an irregularly shaped extraosseous mass extending around the right humerus.

Physical examination revealed a healthy well-developed girl with a tender, palpable, firm mass arising from the proximal shaft of the right humerus. A conventional radiographic image showed an ill-defined, aggressive, osteolytic lesion associated with a partial cortical defect in the proximal shaft of right humerus (Figure 1A). There was no evidence of osteoid or chondroid matrix within the lesion.

Magnetic resonance imaging (MRI) showed an irregularly shaped extraosseous mass extending around the right humerus through the destroyed bone and invading the biceps brachii (Figure 1B, C). The lesion had similar signal intensity to muscle on T1-weighted images and a heterogeneous increase in signal intensity on T2-weighted sequences. A CT-guided biopsy was performed, and the specimen demonstrated atypical cells and reactive new bone. Bone scintigraphy with 740 MBq 99mTc-HMDP showed no abnormal accumulation other than in the proximal right humerus.

**Figure 2:**
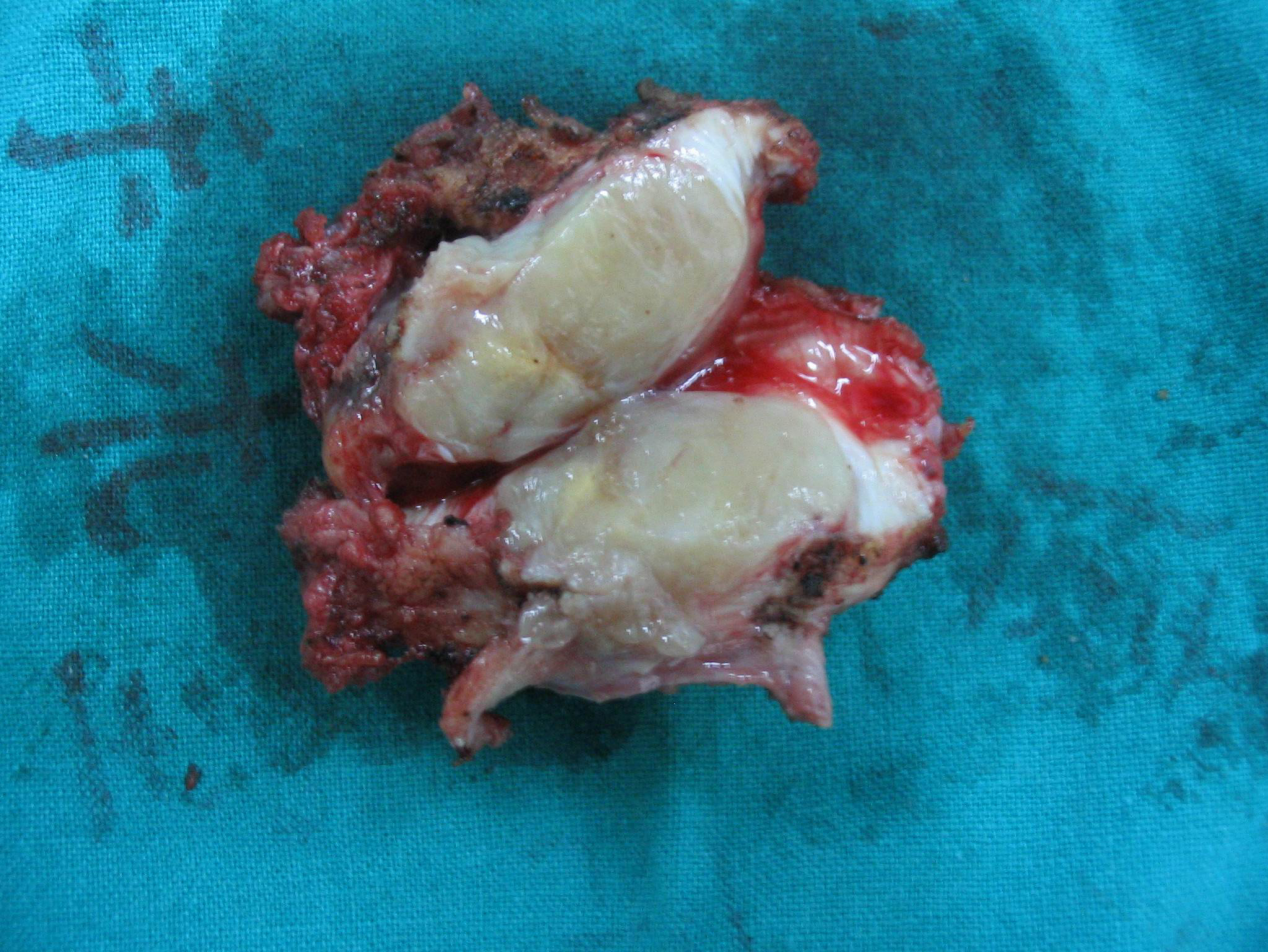
**Gross pathology specimen of the excised tumor mass**. Bone and soft tissue margins were widely free of tumor.

**Figure 3:**
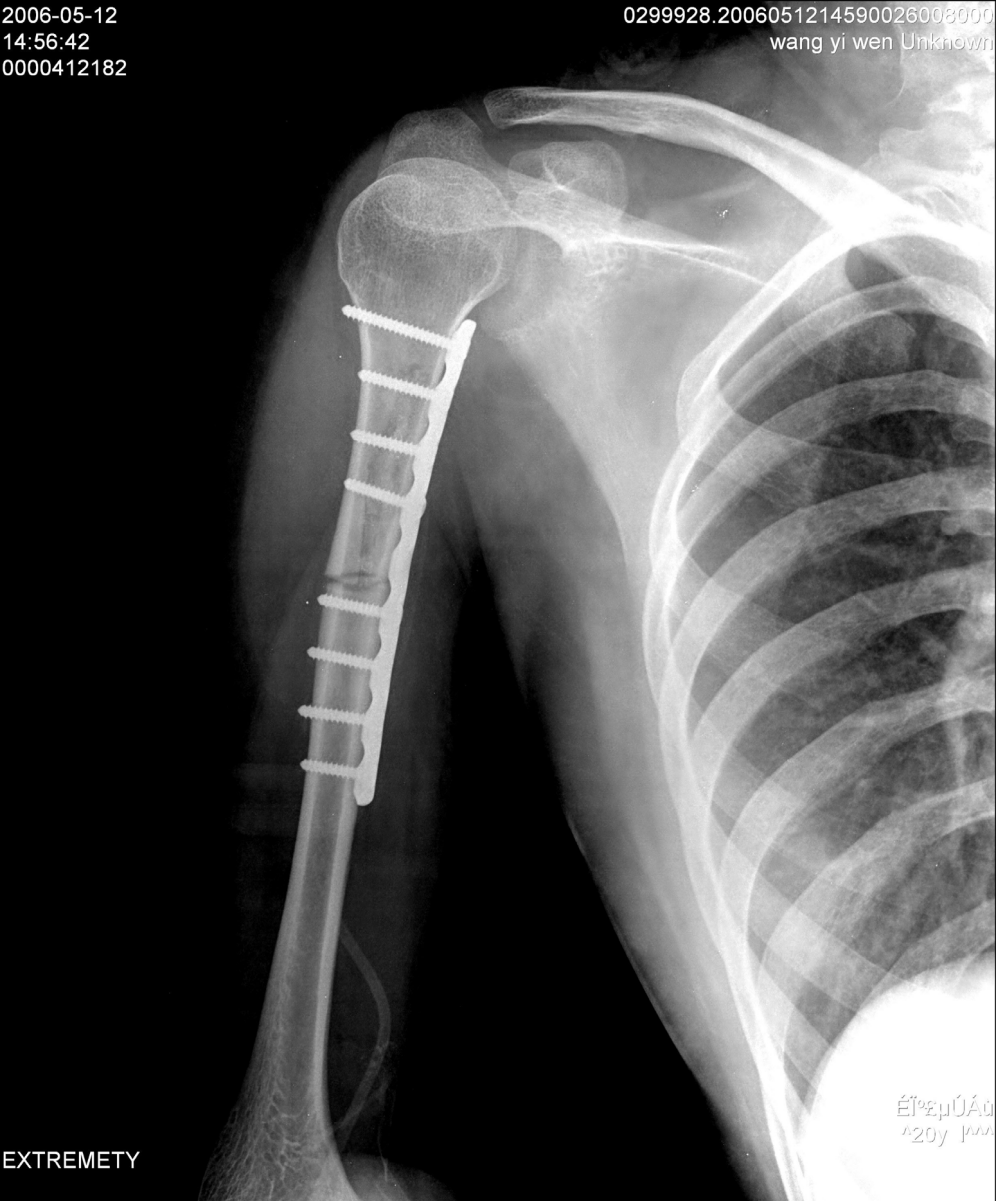
**Radiograph after operation**.

One week after preoperative chemotherapy, which included cisplatin and adriamycin, a total tumor excision-alcoholization-replantation (EAR), internal fixation and bone cement implantation were performed. With the patient under general anesthesia; the anteriomedial approach to the humerus which included the pathway of CT-guided biopsy was used. After detachment of the biceps and triceps brachii, the tumor was found to involve the anteriomedial portion of the proximal humerus and part of the biceps brachii. The site of cortical destruction was not related to the attachment of any tendon or ligament. The whole tumor was detached from the bone and resected. (Figure 2) The humerus was sawed completed through 3 cm distal from the distal end of tumor and a fast frozen biopsy was excised from the periosteum at this site and the proximal end of the tumor with no tumor cells found. The proximal end of the humerus was slivered and the medullary canal was scraped. To destroy all tumor cells that might be remaining in the bone, we put the proximal humerus into 95% ethanol for 30 min. Similar to Pezzillo's method, [5] we then implanted bone cement into the medullary canal and fixed the humerus with a Limited contact dynamic compressive plate (LC-DCP, SYNTHES, Switzerland) (Figure 3).

**Figure 4:**
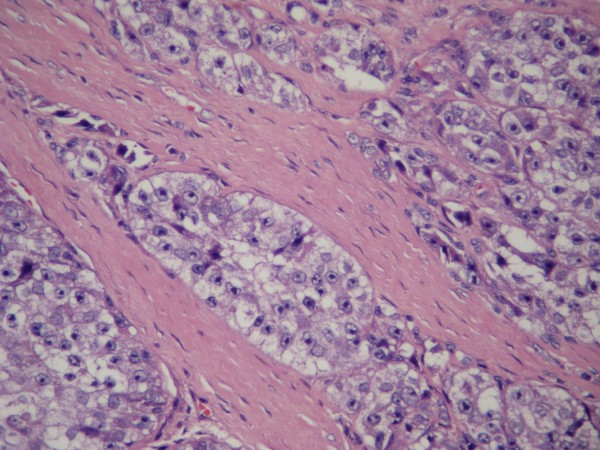
**HE stained photomicrographs showed fibrous septa divided into well-defined nests and polygonal and fusiform cells with clear cytoplasm(×200)**.

**Figure 5:**
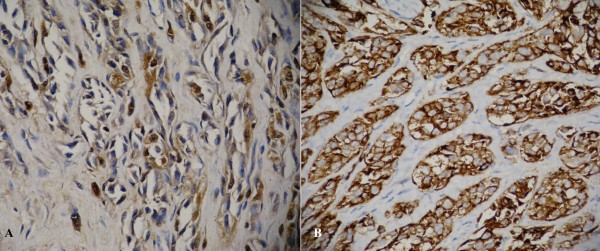
**Immunohistochemistry shows strongly positive immunoreactivity for S-100 protein (×400) (A) and HMB-45 (×400) (B)**.

Routine histopathological studies with hematoxylin-eosin (HE), and immunohistochemical investigations with S-100 and HMB-45 were performed. Histological studies with HE showed fibrous septa divided into well-defined nests and polygonal and fusiform cells with clear cytoplasm (Figure 4). Immunohistochemical studies demonstrated strong positive cytoplasmic staining with S-100 and scattered positivity with HMB-45 (Figure 5). With the tumor displaying these morphological and immunoenzymatic features, the neoplasm was diagnosed as clear cell sarcoma

**Figure 6:**
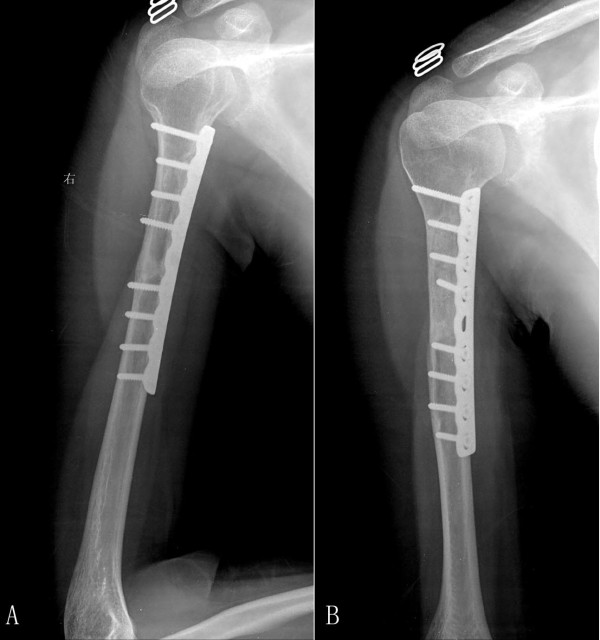
**Radiographs at one year and 28 months after operation**.

After the operation, the patient undertook routine chemotherapy consisting of cisplatin, adriamycin and methotrexate for 2 years. She has been followed up every month and the humerus was found to be completely reconstructed one year after treatment. (Figure 6)

## Discussion

Clear cell sarcoma, also known as malignant melanoma of soft tissue, is a very rare soft tissue neoplasm which comprises 1% of all musculoskeletal sarcomas. It is seen in the extremities in 90-95% of cases. The foot and ankle are the most common primary sites, accounting for 33-43% of cases [6]. It usually presents as a slow-growing mass. Pain and tenderness are the main symptoms in 33-50% of patients [7]. There is a slight female predominance.

The gross appearance of clear cell sarcomas is usually that of a lobular and well-bordered or encapsulated lesion. Microscopically, the tumor in this case consisted of well-defined nests separated by fibrous septa. The tumor cells were polygonal or spindle-shaped and contained eosinophilic or clear cytoplasm. The nuclei had one or two large nucleoli. Some tumor cells may have contained melanin pigment. Unlike clear cell chondrosarcoma, a rare subtype of chondrosarcoma, which presents clear cell chondrocytes, we found no clear cell chondrocytes.

Immunohistochemically, the tumor cells of clear cell sarcomas show strong staining with S-100 protein and HMB-45. All these histological and immunohistochemical features were present in our case. Because the search for metastasis revealed no lesion other than that in the right humerus, we feel that this case represents the extremely rare event of a clear cell sarcoma arising in bone.

Primary clear cell sarcoma and metastatic malignant melanoma represent similar histopathological features. Recent cytogenetic studies have shown that clear cell sarcoma has a t(12;22) (q13;q12) translocation, a feature not encountered in malignant melanoma [8,9]. This chromosomal translocation of clear cell sarcoma has been detected in 50-75% of patients [10]. Cytogenetic analysis was performed in only one of the six previously presented cases of primary clear cell sarcoma of bone and no chromosomal abnormality was detected in that case [11]. We performed cytogenetic analysis and also found no evidence of chromosomal translocation.

The survival rates and metastatic incidence of primary clear cell sarcoma of the bone are unknown because of the limited number of cases reported. The longest survival time of 65 months was reported by Yokoyama et al [2]. Since, at the time our case presented 2 years ago, no specific chemotherapy medicine had been known to treat clear cell sarcoma, we used a routine chemotherapy commonly employed in the case of osteosarcoma including cisplatin, adriamycin and methotrexate. Our patient now has been followed up for two years after the operation, the adjunctive chemotherapy was finished and there have been no findings of metastasis.

At the outset, we suggested to the patient, considering that this is a very malignant tumor, that she should have an amputation at the shoulder. However, the patient and her family wanted her to keep the limb. Therefore, we decided to do a limb salvage operation. We didn't use tumour prosthesis or osteoarticular allograft because the patient wanted to keep the function of her shoulder joint and we found the joint to be normal. As we have used EAR to treat osteosarcoma for many years, and the results were satisfactory[12], we performed a total tumor resection, alcoholization, replantation, internal fixation and bone cement implantation.

The effects of the operation and chemotherapy seem to have been effective, so far, in eradicating the clear cell sarcoma. The patient now feels no discomfort in her right humerus and has no sign of metastasis. We ordered the patient to come in for follow-up every month. Our hope is that this case can be an example of a good outcome of treatment for primary clear cell sarcoma of the bone. We will continue follow up indefinitely.

## Conclusion

We report the first case of primary clear cell sarcoma of humerus. Our findings in this report point out that primary clear cell sarcoma can originate from within the humerus and that limb salvage surgery can achieve good results.

## Consent

Written informed consent was obtained from the patient for publication of this case report and any accompanying images. A copy of the written consent is available for review by the Editor-in-Chief of this journal.

## Competing interests

The authors declare that they have no competing interests.

## Authors' contributions

XL and YD carried out the operation. XL wrote the article. HZ did the pathological study of the case. All authors read and approved the final manuscript.
